# Purulent Pericarditis after Liver Abscess: A Case Report

**DOI:** 10.1155/2014/735478

**Published:** 2014-04-29

**Authors:** María Fidalgo García, Juan Carlos Rodríguez Sanjuán, María Riaño Molleda, Marta González Andaluz, Hector Real Noval, Manuel Gómez Fleitas

**Affiliations:** ^1^General Surgery and Digestive Surgery Department, University Hospital Marqués de Valdecilla, Santander, 39008 Cantabria, Spain; ^2^University of Cantabria, Santander, 39005 Cantabria, Spain

## Abstract

We present the case of a 49-year-old woman, with previous clinical antecedents of recent hepatic metastasis, who was admitted to the ICU due to respiratory failure and hemodynamic instability. She was found to have purulent pericarditis complicated by pericardial tamponade and pleural effusion, as well as surgical site infection, which was the origin of the disease. Cultures of the surgical wound and the pericardial effusion were positive for *Enterococcus faecalis* and *Escherichia coli*. A pericardial tap was performed and the intra-abdominal abscess was surgically drained. Pleural effusion was also evacuated. She received antibiotic treatment and recovered successfully. The only after-effect was a well-tolerated effusive-constrictive pericarditis.

## 1. Introduction

Purulent pericarditis is an uncommon disease, especially after the widespread use of antibiotics. It is defined as an infection of the pericardial space characterized by gross pus in the pericardium or microscopic purulence. Several mechanisms can cause purulent pericarditis, such as direct spread from an adjacent focus of infection, extension from a subdiaphragmatic suppurative focus, or hematogenous spread. Rarely, the primary infectious focus lies in the pericardium [1, 2].

Several cases of acute pericarditis result in or start as a cardiac tamponade. Without an early pericardial drainage and intravenous antimicrobial therapy, this sudden cardiac failure can rapidly lead to death [1, 3].

We report the case of a purulent pericardial effusion that was the extension of an infection from a postoperative subdiaphragmatic suppurative focus.

## 2. Case Presentation

A 49-year-old woman with a personal history of adenocarcinoma of the rectum had been diagnosed 4 years before and treated by resection of the primary tumor, chemotherapy, and left hepatectomy because of metastasis. Ten days before the episode, she had undergone a nonanatomic resection of another liver metastasis between the segments V and VIII. She had been discharged on the postoperative day eight. She was admitted to the emergency department with rapidly progressing dyspnea, orthopnea and edema of the legs.

Physical examination revealed a heart frequency of 110 beats per minute, blood pressure of 85/65 mm Hg, oxygen saturation of 88%, and jugular venous pressure of 22 cm of water. Chest auscultation showed an elevated heart rate and attenuated heart sounds, as well as hypoventilation and lower left rales. She had edema of the legs. In the abdomen, the only pathologic finding was purulent discharge in a Penrose drain.

Laboratory blood tests showed elevation of transaminases (GOT 3904 U/L, GPT 2238 U/L), lactate of 19 mg/dL, C reactive protein of 10.7 mg/dL, and leukocytosis. A chest X-ray demonstrated occupation of the right costophrenic angle.

**Figure 1:**
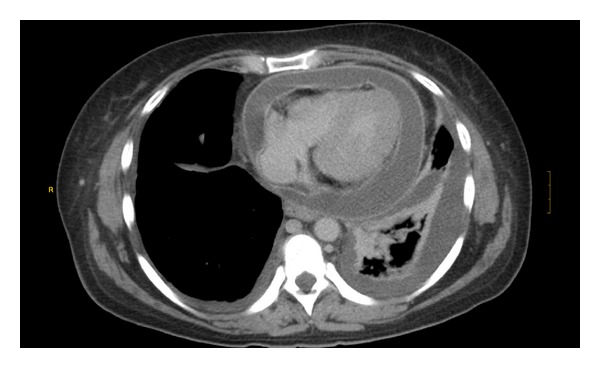
Chest TC, pericardial effusion, and left pleural effusion.

A computerized axial tomography ruled out pulmonary embolism and showed moderate pericardial effusion and an abscess in the liver, at the surgical site, as well as a bilateral pleural effusion Figure 1.

Pericardiocentesis was performed, and 560 mL of straw-colored fluid was removed. Pleural effusion was also evacuated with a chest tube and the patient was referred for surgery in order to drain the intra-abdominal abscess. Broad spectrum intravenous antibiotics were administered.

Pericardial fluid laboratory tests were glucose 2 mg/dL, amylase 27 U/L, LDH 1819 U/L, and proteins 4.9 mg/dL. Cultures were positive for* Enterococcus faecalis *and* Escherichia coli* in the pericardial fluid and in the surgical site abscess and laparotomy wound.

Pleural effusion appeared to be a transudate and no microorganism was isolated on its culture.

A routine transthoracic echocardiography performed 5 days after admission showed a well-tolerated effusive-constrictive pericarditis that was conservatively treated with colchicine, diuretics, and NSAIDs.

The patient recovered and was discharged to the cardiology department eight days after admission.

## 3. Discussion

Purulent pericarditis is defined as a localized infection of the pericardial space characterized by gross pus in the pericardium or microscopic purulence (>20 leukocytes per oil immersion field and/or growth of bacteria in the pericardial fluid culture) [1].

The epidemiology and etiologies of purulent pericarditis have changed significantly in the antibiotic era. In the preantibiotic period, it was a frequent complication of pneumonia, but nowadays its incidence has markedly decreased.

The infectious focus can be localized, such as in pneumonia, mediastinitis, or intra-abdominal abscess, or it can result from hematogenous spread in the context of sepsis.

Patients with a previous pericardial effusion, uremia, immunosuppression, diabetes, cardiac surgery, or chest trauma are more prone to purulent pericarditis [3].

*Staphylococcus aureus *is the most common organism that causes purulent pericarditis. Other frequently isolated bacteria are* Neisseria meningitides *and* Streptococcus pneumoniae. G*ram-negative rods,* Pseudomonas aeruginosa, Salmonella,* anaerobes, and fungal pathogens are less common.

The clinical diagnosis is difficult because some features are unspecific and can be blamed on the underlying infectious process (fever, dyspnea, and tachycardia) and some other features that are more specific are often absent (pleuritic pain, pericardial friction rub). This can lead to a delayed diagnosis, only made when the patient is already hemodynamically instable.

Sagrista-Sauleda et al. [1] compared a series of patients with the diagnosis of pericardial effusion and found that 80% of them presented with cardiac tamponade. He also found, nevertheless, that purulent pericarditis was the underlying cause of this syndrome in a minority of the cases [4].

That is why an echocardiogram should be obtained if the suspicion of pericardial involvement by an underlying infectious process arises, since it can rapidly turn into a critical situation. The echocardiogram can show signs of cardiac tamponade (right chambers collapse, changes in mitral, or tricuspid flow) and guide a pericardial tap, which can be diagnostic or therapeutic. Computerized axial tomography is also a useful diagnostic aid, as it was in our case.

Definite diagnosis requires laboratory tests of the pericardial fluid (LDH, PMN, and glucose) as well as cytology, Gram, and cultures for ordinary organisms, anaerobes, and fungi.

Treatment consists on drainage of the pericardial fluid, and culture guided antibiotics for 4 to 6 weeks [2]. Current guidelines recommend surgical drainage in the case of purulent pericarditis [5, 6]. However, our patient had undergone major surgery just a few days before, and, since pericardiocentesis caused a marked improvement of her overall status, an agreement was reached with heart surgeons and ICU physicians that surgery ought to be avoided if possible. In some cases a constrictive pericarditis develops during the initial phase of the disease [1], as happened with our patient. Again, guidelines state that, in the case of constriction that remains patent after 2 years, a pericardiectomy should be done [6]. Luckily, pericardiocentesis and antibiotherapy caused complete resolution of the infection, the effusive-constrictive pericarditis was well tolerated, and a pericardiectomy could be spared.

Our case report is one of the few examples of purulent pericarditis after abdominal surgery that has been reported in the medical literature. In the study of 33 patients with purulent pericarditis by Sagrista-Sauleda et al., an intra-abdominal focus occurred in 19% of the infections, and only two patients had undergo intra-abdominal surgery (cholecystectomy after gallbladder empyema and sepsis after hepatic transplantation). The case reported by Laínez et al. [2] was caused by an abscessified hydatid cyst that caused a disruption of the diaphragm and communicated with the pericardium. It resolved with pericystectomy and cardiac surgery.

De Souza Paolino et al. [7] and Devin and Merdinger [8] suggest that a communication between the peritoneum and the pericardium might arise during the fourth month in the ontogenic development of the fetus, because of embryologic defects in diaphragm closure.

In conclusion, purulent pericarditis is a rare but potentially fatal complication after abdominal surgery and a high suspicion index is needed in patients who develop cardiac symptoms in the context of a surgical site infection. Early diagnosis by means of a good physical examination and echocardiography or computerized axial tomography as well as an immediate treatment with pericardial tap and intravenous antibiotherapy guided by the bacteriological studies can avoid this critical situation that otherwise results in patient death.
